# Did you rule out neurosyphilis?

**DOI:** 10.1590/S1980-57642010DN40400014

**Published:** 2010

**Authors:** Ricardo Nitrini, Anderson Rodrigues Brandão de Paiva, Leonel Tadao Takada, Sonia Maria Dozzi Brucki

**Affiliations:** 1-4 MD, PhD, Behavioral and Cognitive Neurology Unit, Department of Neurology, and Cognitive Disorders Reference Center (CEREDIC), Hospital das Clínicas of the University of São Paulo School of Medicine, São Paulo SP, Brazil.

**Keywords:** neurosyphilis, syphilis, dementia, neuropsychiatry, paretic neurosyphilis

## Abstract

Neurosyphilis, formerly a frequent cause of dementia, is now a rare condition in developed countries. However, syphilis remains common in many developing countries, where adequate diagnosis and treatment of early syphilis may be lacking, increasing the chances of neurosyphilis and prevalence of syphilitic dementia.

**Objectives:**

To present cases of syphilitic dementia seen in a cognitive and behavioral neurology unit in Brazil, emphasizing their first symptoms and the challenges they posed in diagnosis.

**Methods:**

At our unit of the Hospital das Clínicas of the University of São Paulo, all patients are submitted to blood treponemal tests. When the test is positive, a lumbar puncture is performed. We retrospectivelly reviewed all cases of neurosyphilis seen in our unit from January 1991 to November 2009.

**Results:**

Nine cases of neurosyphilis (0.77% of the 1160 cases in our files) were identified over the period. Patients with neurosyphilis were all men, had a mean age of 47.8 (±13.0) years (median of 43 years), and presented with various neuropsychiatric syndromes and elusive diagnoses. The median time from onset of symptoms to diagnosis was 24 months and only one patient made a full recovery after treatment.

**Conclusions:**

Neurosyphilis is not frequent but remains present, causing several types of neuropsychiatric syndromes. As it is very simple to rule out neurosyphilis by performing a blood treponemal test, this test should be performed in all patients with neuropsychiatric symptoms, particularly in regions of the world where syphilis is still a commonly occurring disease.

## Abstract (pt)

Neurossífilis, anteriormente uma causa freqüente de demência, é atualmente rara nos países desenvolvidos. A sífilis é ainda uma doença comum em muitos países em desenvolvimento, onde o diagnóstico e tratamento da sífilis precoce podem não ser adequados, o que aumenta a possibilidade de ocorrência de neurossífilis e de demência.

**Objetivos:**

apresentar casos de demência sifilítica atendidos em uma unidade de neurologia cognitiva e do comportamento no Brasil, enfatizando os primeiros sintomas e os desafios que impuseram ao diagnóstico.

**Métodos:**

Em nossa unidade do Hospital das Clínicas da Universidade de São Paulo, todos os pacientes são submetidos a teste treponêmico no sangue. Quando o teste é positivo, é realizada punção lombar. Avaliamos retrospectivamente todos os casos de neurossífilis atendidos em nossa unidade de janeiro de 1991 a novembro de 2009.

**Resultados:**

Nove casos de neurossífilis (0,77% dos 1.160 casos de nossos arquivos) foram identificados neste período. Os pacientes com neurossífilis eram todos homens, com idade média de 47,8 (±13,0) anos (mediana de 43 anos), e apresentaram-se com vários tipos de síndromes neuropsiquiátricas, de difícil diagnóstico. O tempo médio entre o início dos sintomas e o diagnóstico foi de 24 meses e apenas um paciente teve recuperação completa após o tratamento.

**Conclusões:**

Neurossífilis não é frequente, mas ainda está presente causando vários tipos de síndromes neuropsiquiátricas. Como é muito simples excluir o diagnóstico de neurossífilis mediante teste treponêmico no sangue, este teste deve ser realizado em todo paciente com sintomas neuropsiquiátricos, particularmente nas regiões do mundo onde a sífilis é ainda uma doença comum.

Neurosyphilis was probably the most common cause of dementia until the mid-twentieth century, because syphilis was very frequent and there was no adequate treatment for early syphilis. Thus, a proportion of cases evolved to late forms, when dementia of neurosyphilis may occur. On the other hand, life expectancy was low, which meant that the degenerative dementias that are more frequent after 65 years were much rarer than today. The advent of penicillin changed this scenario, because penicillin is highly effective in the treatment of early syphilis, limiting the spread of the disease and preventing progression to late forms.^1^

Amongst these late forms, dementia may occur in meningovascular neurosyphilis, which may cause vascular dementia or hydrocephalus by blocking the transit of cerebrospinal fluid (CSF). But the most frequent cause of syphilitic dementia is paretic neurosyphilis (or *dementia paralytica*, or general paralysis of the insane or general paresis). Paretic neurosyphilis occurs in about 5% of patients untreated for early syphilis, usually from 10 to 25 years after the infection.^2,3^

In paretic neurosyphilis there is a chronic meningoencephalitis caused by the invasion of brain parenchyma by *Treponema pallidum*. The most conspicuous findings are cortical atrophy, most markedly in the anterior portions of the brain, thickening of the meninges and enlargement of the ventricles. There may be strong lymphoplasmocytic infiltrates in the meninges and its vessels, which accumulate forming a cuff in the perivascular spaces of the brain. Variable degrees of proliferation of the intimal layer with narrowing and occlusion of the vessel lumen are seen in small capillaries and venules, which are probably responsible for the marked loss of neurons, astrogliosis and microglial cells that occur in the cerebral cortex, cerebellum and basal ganglia.^2^ In other cases, the lymphoplasmocytic infiltrate is minor and the degenerative changes predominate with more severe cortical atrophy.^4^ Treponemes may be evident in the cortex in 25 to 40% of the cases,^5^ and are more frequent in those cases where the lymphoplasmocytic infiltrate is minor.^4^

The efficacy of penicillin in the treatment of early syphilis reduced the frequency of dementia caused by neurosyphilis in developed countries in such a way that in 2001 the American Academy of Neurology guidelines for the diagnosis of dementia proposed that screening for syphilis in all patients with dementia was no longer necessary.^6^ Dementia caused by neurosyphilis has become so uncommon in the developing world that single cases are published as case reports of rare causes of dementia, sometimes with challenging diagnosis because neuropsyphilis is not regularly included amongst diagnostic hypotheses.^7-9^

In Brazil, as well as in many other developing countries, syphilis is still a common disease,^10^ and adequate diagnosis and treatment of early syphilis may be lacking, making syphilitic dementia more frequent. In a multicenter study in Brazil, the prevalence of syphilis in patients with mental illness was 1.12%.^11^ The Brazilian Academy of Neurology recommends screening for syphilis in the diagnosis of dementia through use of blood tests.^12^

Diagnosing syphilitic dementia demands a considerable degree of suspicion, particularly in the initial phase, when treatment is more effective. When dementia is severe, the chances of full recovery is low.^13^

Our aims in this retrospective study were to present cases of patients with syphilitic dementia assisted in a cognitive and behavioral neurology unit in São Paulo, Brazil, emphasizing their first symptoms and the challenges they posed in diagnosis.

## Methods

At the Behavioral and Cognitive Neurology Unit of the Hospital das Clínicas of the University of São Paulo, a public hospital, all patients are submitted to blood tests for syphilis, including a treponemal test. When the treponemal test is positive, a lumbar puncture is performed, and the diagnosis of neurosyphilis is confirmed by positive VDRL and treponemal test in CSF. When only the treponemal test is positive in the CSF, the diagnosis of neurosyphilis is still possible when there is pleocytosis (≥5 cells/mm^3^) and/or slightly elevated protein levels (45-200 mg/dL) with a high gammaglobulin proportion (>14%). All patients with syphilis are tested for HIV infection.

We identified all patients with behavioral or cognitive disturbances caused by neurosyphilis that were seen in our unit from January 1991 to November 2009.

The cases of neurosyphilis were analyzed with respect to demographic characteristics, main symptoms and signs, duration of symptoms until diagnosis, neuropsychological profile and exams. The classification of the forms of neurosyphilis into general paresis, taboparesis or meningovascular syphilis was based on guidelines proposed by Merritt et al.^2^ All patients were treated with intravenous penicillin at high doses and treatment outcomes were evaluated.

This study was approved by the Research Ethics Committee of the Hospital das Clínicas of the University of São Paulo, Brazil.

## Results

In this 19-year period, 1,160 patients (54.6% women) presenting cognitive or behavioral disturbances, with a mean age of 68.3 (±11.6) years and 5.6 (±4.6) years of schooling, were assisted at our unit. Nine cases of neurosyphilis (0.77%) were identified in this period. Patients with neurosyphilis were all men, had a mean age of 47.8 (±13.0) years (median of 43 years), and 8.8 (±6.6) years of schooling (median of 8 years of schooling). Demographic data and diagnoses are shown in Table 1. Median time to diagnosis from onset of symptoms was 24 months. Seven cases were diagnosed as paretic neurosyphilis, one with taboparesis (paretic neurosyphilis with tabetic signs) and one case as meningovascular neurosyphilis.

**Table 1:** Demographic data, diagnoses and time to diagnosis.

Case/Year	Gender	Age	Education(years of schooling)	Diagnosis (clinical syndrome)	Time to diagnosis (months)
1/1991	M	32	15	PN	36
2/1993	M	61	1	PN	17
3/1994	M	43	15	PN	12
4/1997	M	42	4	MV	24
5/1997	M	48	8	PN	12
6/1995	M	36	5	TP	6
7/2000	M	59	16	PN	24
8/2000	M	39	16	PN	48
9/2003	M	71	0	PN	36

MV: meningovascular neurosyphilis; PN: paretic neurosyphilis; TP: taboparesis.

One patient (case 4) reported a history of syphilis. The neurological examination disclosed hyperactive deep reflexes in six cases (cases 1,2,3,4,5,9) dysarthria in four cases (cases 1,3,4,8), hemiparesis in three (cases 2,5,6), and absence of the light reflex with an active reaction in accommodation (Argyll Robertson pupil) in two cases (cases 1, 6). One patient (case 9) had parkinsonian signs. Beyond the cognitive and behavioral abnormalities, the neurological examination did not disclose other signs in one patient (case 7), while subtle signs were seen in cases 1 and 8.

Serum and CSF tests are shown in Table 2. All patients had positive non-treponemal tests (VDRL or Wassermann’s test) in the CSF and eight presented with pleocytosis.

**Table 2:** First blood tests and cerebrospinal fluid (CSF) examinations.

Case	Serum			CSF				
	VDRL	Trep. T.		Leukoc/mm^3^	Protein (mg/dl)	Gammagl. (%)	VDRL	TPHA
1	1/8	TPHA + (>1/1024)		13	76	49.7	1/8	>1/1024
2	1/16	FTA-Abs +		10	64	20.3	1/4	>1/1024
3	NA	FTA-Abs +		58	50	26.3	WR 1/4	1/512
4	1/1	FTA-Abs +		36	98	31.3	1/4	+
5	NA	FTA-Abs +		5	60	42.6	+	+
6	1/8	TPHA + (1/256)		3	33	35.9	1/2	1/256
7	1/32	FTA-Abs +		165	127	31.8	WR+	1/512
8	NR	ELISA +		45	70	39.6	+	1/512
9	1/256	TPHA +		133	206	33.9	1/128	+

ELISA: Enzyme linked immunoassay; FTA-Abs: fluorescent treponemal antibody-absorbed; Gammagl.: gammaglobulin; Leukoc: leukocytes; NA: not available; NR: Non reactive; WR: Wassermann's reaction; TPHA: Treponema pallidum haemagglutination assay.

Table 3 contains a summary of the initial symptoms, the presumptive or first diagnostic hypothesis, and outcome after treatment with intravenous penicillin from 18 to 24 million IU for 10 to 21 days. Only one case made a full recovery, returning to his previous professional activities. In most cases there was a partial recovery or the condition was considered to be unchanged when the progression of the disease was halted without significant improvement.

**Table 3:** Initial symptoms, first diagnostic hypothesis, Mini-Mental State Exam (MMSE) and outcome.

Case	Initial symptoms	First diagnosticHypothesis	Initial MMSE score	Outcome	Highest MMSE score after treatment
1	Persecutory delusions, auditory hallucinations and epileptic seizures	Psychosis, schizophrenia	23	Unchanged	22
2	"Meningoencephalitis", memory decline, topographical disorientation	Herpetic encephalitis	NA	Unchanged	19
3	Irritability, motor and psychic slowness	"Organic" disorder	11	Partial improvement	29
4	Tremors, seizures, memory decline	Cerebrovascular disease	17	Partial improvement	23
5	Forgetfulness, dysexecutive syndrome, seizures	Dementia not specified	25	Unchanged	25
6	Agressivity, memory decline, collecting behavior	Dementia not specified	0	Partial improvement	25
7	Memory decline, topographical disorientation, social inadequacy	Alzheimer's disease	22	Full recovery	27
8	Irritability, social withdrawal, dysexecutive syndrome	Frontotemporal dementia	29	Unchanged	29
9	Delirium, tremors, memory decline, topographical disorientation	Dementia with Lewy bodies	10	Partial improvement	13

Table 4 summarizes neuroimaging data. Vignettes of the nine cases are presented in the appendix.

**Table 4:** Neuroimaging findings.

Case	Computed tomography (CT) Magnetic resonance imaging (MRI)	Single Photon computed tomography (SPECT)
1	NA	Slight hypoperfusion in the left fronto-parietal cortex
2	CT: Normal	
3	MRI: Cortical atrophy, more severe in the right hemisphere	NA
4	CT: Multiple infarcts (larger infarct in the right temporoparietal area)	NA
5	MRI: Diffuse cortico-subcortical atrophy and infarct in the right temporal lobe	NA
6	CT: Diffuse cortico-subcortical atrophy	NA
7	CT: Diffuse cortico-subcortical atrophy	NA
8	MRI: Diffuse cortico-subcortical atrophy	Slight hypoperfusion in the frontal cortex.
9	MRI: Diffuse cortico-subcortical atrophy	NA

NA: not available.

## Discussion

Our findings show that syphilitic dementia still occurs in our country, albeit at a much lower frequency compared to levels reported before penicillin became available.^2^

All patients in this series were men. Syphilis, and particularly neurosyphilis, has always been more frequent in men since its first descriptions. In the past, syphilis was considered to be more common in men because they were infected by women prostitutes, with many men being infected by a few prostitutes.^14^ More recently, syphilis has been more frequent in men who have sex with other men.^15^ Syphilis and neurosyphilis have also been more frequent in HIV+ patients.^16^ In this series, none of the patients were HIV+, probably because there is a referral bias, with HIV+ patients being assisted in other departments of our hospital.

There are several aspects that need to be stressed. Most of our patients were younger than 50 years old and can be classified as young-onset dementia.^17^ The time from the initial symptoms to the diagnosis of neurosyphilis (median of 24 months) was very long. This may be in part explained by the protean early symptoms. The presumptive diagnoses were diverse, ranging from schizophrenia to herpetic encephalitis, with many cases being attributed to degenerative or unspecified causes. Neurosyphilis has been long recognized as a “great imitator”,^18^ and the clinical diagnosis can be very difficult because “pure psychiatric cases” may be found or other diagnoses may be entertained.

Previous history of syphilis is rarely obtained in these cases, and in this study, only one patient reported a history of syphilis. The neurological examination may show hyperactive deep reflexes, hemiparesis, dysarthria, pupillary abnormalities and absence of the light reflex, and even parkinsonian signs. In this series, “somatic” neurological examination was normal in only one patient, with only subtle signs in two.

Neuroimaging, the most important subsidiary exam for the diagnosis of dementia, may be misleading in paretic neurosyphilis because the diffuse atrophy may be suggestive of degenerative dementia.^19^ In this small series, five cases had CT or MRI showing diffuse cortico-subcortical atrophy without other findings.

In a study performed in New Orleans, Louisiana, USA, the authors reviewed all cases of neurosyphilis seen in a university hospital from 1998 to 2001. Cases with positive-HIV serology were excluded. They found 17 cases of neurosyphilis, with mean age of 49.7 (±12.0) years. Neuropsychiatric symptoms were frequent (four presented with violent behavior, six were disoriented, and four had memory deficits).^20^

As evidenced, neurosyphilis is not a rare disease, but its diagnosis may be very elusive. However, it is easy to rule out this diagnosis using blood tests. Serum VDRL may be non reactive in neurosyphilis, but a negative treponemal test (FTA-Abs: fluorescent treponemal antibody-absorbed or TPHA: *Treponema pallidum* haemagglutination assay or ELISA: Enzyme linked immunoassay) virtually excludes syphilis.^19^ If serum treponemal antibody test is reactive in a suspected case of neurosyphilis, lumbar puncture is mandatory. A non-reactive treponemal test in the CSF virtually excludes neurosyphilis. Although the CSF-treponemal tests have high sensitivity, their specificity is not high, as a reactive test may be due to diffusion of antibodies from the serum. When a CSF-treponemal test is reactive, the diagnosis of neurosyphilis is supported by reactive CSF-VDRL, which is highly specific, though false reactive CSF-VDRL may occur when there is blood in the CSF or when the blood-brain barrier is severely breached. However, as its sensitivity is relatively low, false negative CSF-VDRL tests may occur in neurosyphilis.^16^, ^19^ When the CSF treponemal test is reactive and the CSF-VDRL is non-reactive, diagnosis of neurosyphilis is supported by pleocytosis (≥5 cells/mm^3^), slightly elevated protein (45-200 mg/dL) with elevated CSF-IgG. The diagnosis may be particularly difficult in patients with HIV-1 infection because slight pleocytosis and elevated protein in the CSF are also common in this condition. Given that syphilis is common in HIV-infected individuals, a reactive FTA-Abs test in the CSF could be caused by diffusion from the serum, in the absence of neurosyphilis. If a patient with possible paretic neurosyphilis has a reactive CSF-treponemal test and pleocytosis or elevated protein concentration, even if the CSF-VDRL is negative, treatment for neurosyphilis should be given.^16,19^ This is particularly relevant for patients with HIV-1 infection.^16^

In our cases, serum VDRL was non-reactive in one case whereas serum and CSF treponemal tests were reactive in all patients. CSF-VDRL was reactive in all cases in which it was performed (7 cases). In the other two patients, VDRL was substituted by the Wassermann’s complement fixation test, which was reactive in both cases (Table 2). Pleocytosis was present in seven cases and high concentration of protein in the CSF in eight cases. A very high proportion of gammaglobulins (>30%), which are mostly IgG, was found in seven cases. High levels of IgG is a frequent finding in neurosyphilis, particularly in paretic dementia.^21^

All patients were treated with intravenous penicillin G for 10 to 21 days. In our service we treat patients with severe forms of neurosyphilis with 24 million international units of penicillin G for 21 days. In this series, a few patients that came to our service after recent treatment had received intravenous penicillin for 10 to 14 days. All patients were followed-up with lumbar puncture repeated at 6-month intervals for 2 to 3 years. No cases had persistent pleocytosis, and no patients were re-treated with antibiotics for neurosyphilis.

In neurosyphilis, significant clinical improvement occurs mainly when the treatment is administered in the first few months after the onset of the mental disturbances, particularly in mild and moderate disturbances.^2,13^ In our series however, only one patient returned to his previous professional activity whereas most patients became dependent on others or were able to work only in less demanding activities. This may be explained by the long delay reaching diagnosis, in a condition where the diagnosis is so simple, when only a blood treponemal test need be performed.

The main conclusions of this paper are:

[i] neurosyphilis is not frequent but it is still present, causing many types of neuropsychiatric syndromes, from schizophreniform psychotic syndrome to dementia mimicking Alzheimer’s disease;
[ii] as it is very simple to rule out neurosyphilis by performing a blood treponemal test, this test should be performed in all patients with neuropsychiatric symptoms, particularly in regions of the world where syphilis is still a common disease.
